# Spectral scanning and fluorescence lifetime imaging microscopy (FLIM) enable separation and characterization of *C. elegans* autofluorescence in the cuticle and gut

**DOI:** 10.1242/bio.060613

**Published:** 2024-12-30

**Authors:** Heino J. Hulsey-Vincent, Elizabeth A. Cameron, Caroline L. Dahlberg, Domenico F. Galati

**Affiliations:** Western Washington University, Bellingham, WA, USA

**Keywords:** *C. elegans*, Autofluorescence, GFP, FLIM, Spectral, Gut granules

## Abstract

*Caenorhabditis elegans* gut and cuticle produce a disruptive amount of autofluorescence during imaging. Although *C. elegans* autofluorescence has been characterized, it has not been characterized at high resolution using both spectral and fluorescence lifetime-based approaches. We performed high resolution spectral scans of whole, living animals to characterize autofluorescence of adult *C. elegans*. By scanning animals at 405 nm, 473 nm, 561 nm, and 647 nm excitations, we produced spectral profiles that confirm the brightest autofluorescence has a clear spectral overlap with the emission of green fluorescent protein (GFP). We then used fluorescence lifetime imaging microscopy (FLIM) to further characterize autofluorescence in the cuticle and the gut. Using FLIM, we were able to isolate and quantify dim GFP signal within the sensory cilia of a single pair of neurons that is often obscured by cuticle autofluorescence. In the gut, we found distinct spectral populations of autofluorescence that could be excited by 405 nm and 473 nm lasers. Further, we found lifetime differences between subregions of this autofluorescence when stimulated at 473 nm. Our results suggest that FLIM can be used to differentiate biochemically unique populations of gut autofluorescence without labeling. Further studies involving *C. elegans* may benefit from combining high resolution spectral and lifetime imaging to isolate fluorescent protein signal that is mixed with background autofluorescence and to perform useful characterization of subcellular structures in a label-free manner.

## Teaser

**Summary:** This paper describes how spectral scanning and FLIM can be combined to characterize autofluorescence and improve GFP quantification in autofluorescent tissues.

## INTRODUCTION

The model organism, *Caenorhabditis elegans* (*C. elegans*), is a transparent nematode that is amenable to microscopy and study through live imaging. *C. elegans* imaging often uses fluorescence to analyze promoter reporters, fusion proteins or dyes that label subcellular structures (Corsi et al., 2015; El Mouridi et al., 2022; Mendoza et al., 2024; Yemini et al., 2021). Fluorescence imaging of *C. elegans* is central to diverse research questions from developmental biology to behavioral neurobiology (Bao et al., 2006; Chung et al., 2013; Tian et al., 2009). However, fluorescence imaging in *C. elegans* must contend with autofluorescence emitted from tissues and materials, such as a protective cuticle and intestinal lysosome-related organelles (gut granules) (Hermann et al., 2005; Pincus et al., 2016; Teuscher and Ewald, 2018). This was observed as early as the first account of *C. elegans* expressing GFP, where autofluorescence was noted to obscure the GFP signal (Chalfie et al., 1994). Methods that overcome autofluorescence in *C. elegans* will remove barriers to fluorescence imaging in live animals. This is especially true in areas and tissues where autofluorescence is particularly strong, such as the gut and cuticle (Heppert et al., 2016; Komura et al., 2021; Pincus et al., 2016).

Spectral approaches are a common way to overcome *C. elegans* autofluorescence. For example, carefully chosen bandpass filters can partially separate autofluorescence emission from GFP emission in the gut (Morris et al., 2018), intensity-based autofluorescence correction can improve the GFP signal to noise ratio in the developing embryo (Rodrigues et al., 2022), and spectral unmixing can separate fluorescent protein emission from autofluorescence (Jones and Ashrafi, 2009). Alternatively, one can rationally choose fluorescent proteins that have minimal spectral overlap with autofluorescence (Heppert et al., 2016; Thomas et al., 2019) or use non-genetically encoded fluorescent probes that emit in the infra-red range (Hendler-Neumark et al., 2021; Rashtchian et al., 2021). Finally, studies have used the genetic power of *C. elegans* to remove the source of autofluorescence by performing experiments in backgrounds that do not produce autofluorescent gut granules (Eichel et al., 2022).

An additional parameter that can differentiate spectrally similar fluorophores is fluorescence lifetime, which is the temporal delay between the arrival of an excitation photon and the generation of an emission photon. Each fluorophore has a unique fluorescence lifetime that depends upon the chemical structure of the fluorophore and the environment (i.e. solvent) that surrounds the fluorophore (as reviewed in Datta et al., 2020). Fluorescence lifetime can be imaged with fluorescence lifetime imaging microscopy (FLIM) and quantified through curve fitting or phasor analysis (Phasor-FLIM). Although curve fitting is widely accepted, this approach requires pre-existing knowledge about the decay parameters of the fluorophores that are being analyzed. In contrast, Phasor-FLIM analysis does not make any assumptions about the underlying decay parameters of fluorophores (as reviewed in Malacrida et al., 2021). Phasor-FLIM has been used to quantify NADH/NAD(P)H and FAD/FADH_2_ ratios in metabolic studies (Bhattacharjee et al., 2017; Ma et al., 2016), separate spectrally similar fluorophores (Gonzalez Pisfil et al., 2022), distinguish known fluorophores from autofluorescence (Szmacinski et al., 2014), and quantify fluorescence resonance energy transfer (FRET) efficiency of fluorescent proteins (Lou et al., 2019). In *C. elegans*, FLIM has been used to investigate protein–protein interactions (Gallrein et al., 2021; Laine et al., 2019; Llères et al., 2017) and environmental effects on metabolic dyes (Chen et al., 2023). However, these studies do not address native autofluorescence in *C. elegans*, which has both biological relevance and a long history of complicating fluorescent protein quantification.

In this study, we performed a systematic analysis of the spectral and lifetime properties of *C. elegans* autofluorescence relative to the emission profiles of conventional fluorophores, such as GFP and mCherry. We show that dim GFP fluorescence can be reliably separated from bright cuticle autofluorescence using Phasor-FLIM. We also demonstrate that spectrally similar gut autofluorescence can be characterized in a label-free manner by capitalizing on heterogeneous lifetimes.

## RESULTS AND DISCUSSION

**Fig. 1.**
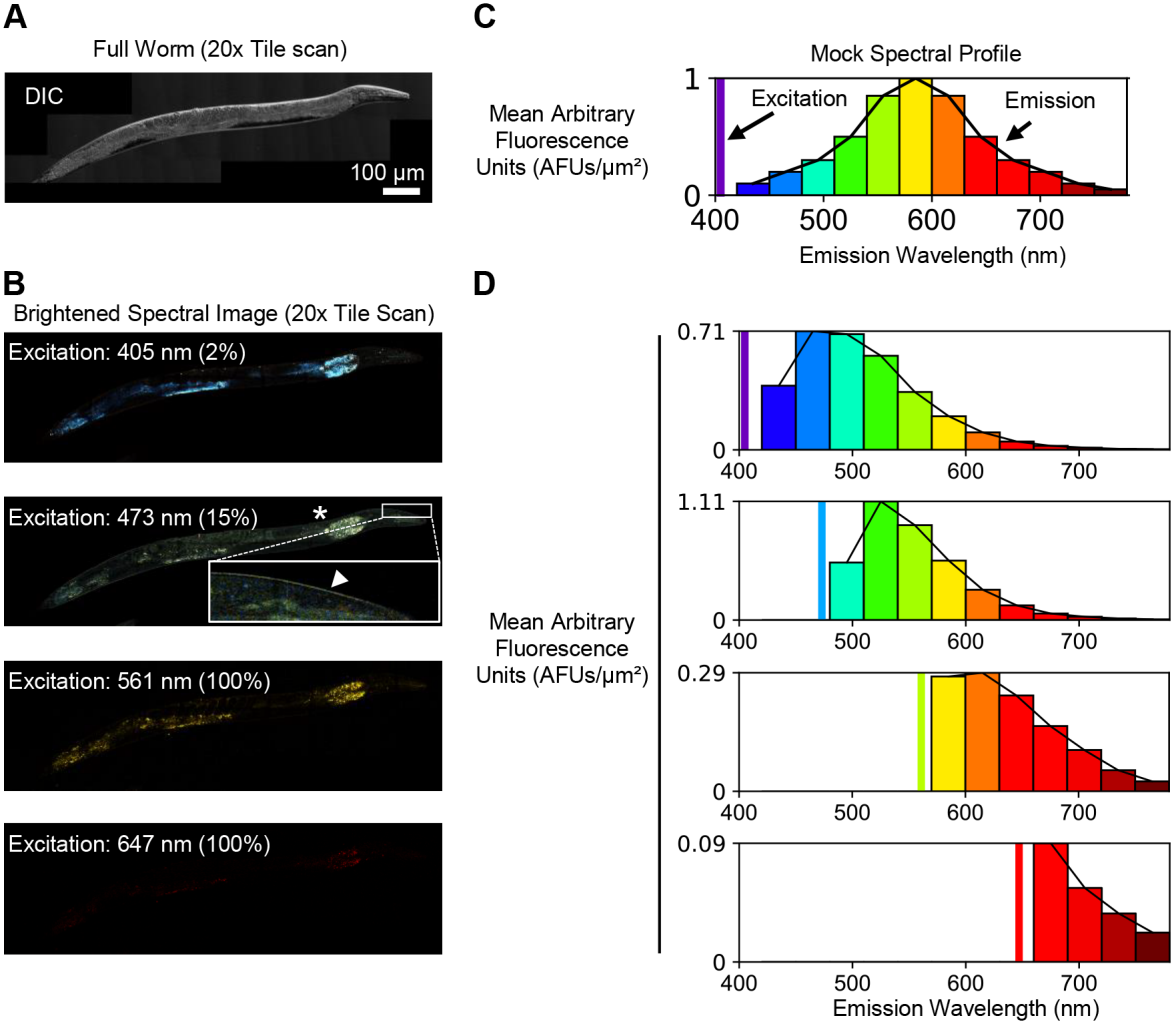
***C. elegans* autofluorescence is most prominent at shorter wavelengths.** (A) An image of a 3-day-old *C. elegans* acquired using differential interference contrast (DIC). (B) Colorized spectral images of a single plane in the same animal in A showing autofluorescence emission after excitation with the following laser lines: 405 nm (emission 420-780 nm), 473 nm (emission 480-780 nm), 561 nm (emission 570-780 nm), and 647 nm (emission 660-780 nm). Relative laser power is reported as a percentage. Regions of gut (asterisk) and cuticle (box and arrowhead) autofluorescence are distinguished in the 473 nm excitation image. (C) A mock spectral profile of *C. elegans.* The excitation wavelength is represented by a vertical bar (purple). The emission was collected in 30 nm bins and plotted as mean arbitrary fluorescence units (AFU) per µm^2^. (D) The spectral profiles for each colorized spectral image in panel (B). See Materials and Methods and Fig. S1 for detailed explanation of how images were colorized and converted to spectral profiles. Similar spectral profiles were generated for five additional independent animals during two imaging sessions (presented in Fig. S2).

To determine the autofluorescence spectrum of live *C. elegans*, young adult animals were stimulated with four common excitation lines [405 nm (BFP/DAPI), 473 nm (GFP), 561 nm (mCherry), and 647 nm (emiRFP670/Alexa 647)] and non-overlapping 30 nm emission bins were collected across the entire visible and near infrared spectrum (Fig. 1A,B, Fig. S1). To quantify the spectral data, the mean pixel intensity for each emission bin was calculated across the entire animal and plotted as a spectral profile (Fig. 1C,D). In agreement with other findings (Heppert et al., 2016; Hermann et al., 2005), this approach revealed that 405 nm and 473 nm excitation stimulate autofluorescence with strong emission in the 450-600 nm range (Fig. 1B,D). Conversely, 561 nm excitation stimulates autofluorescence with weak emission in the 570-720 nm range, and 647 nm excitation produces little to no emission (Fig. 1B,D). To assess the variability of the spectral profiles, we collected data from five separate animals. We found that 405 nm and 473 nm excitation consistently stimulate strong emission (Fig. S2A-E), while 561 nm excitation produced weak and variable emission that ranged from barely detectable (Fig. S2A-C) to undetectable (Fig. S2D,E). Anatomically, the strongest autofluorescence was observed in the gut (Fig. 1B, asterisk) and cuticle (Fig. 1B, box and arrowhead). These results demonstrate that *C. elegans* produce a wide spectrum of autofluorescence that is distributed throughout the body of the animal, and the most intense emission overlaps with commonly used green fluorescent proteins and dyes.

**Fig. 2.**
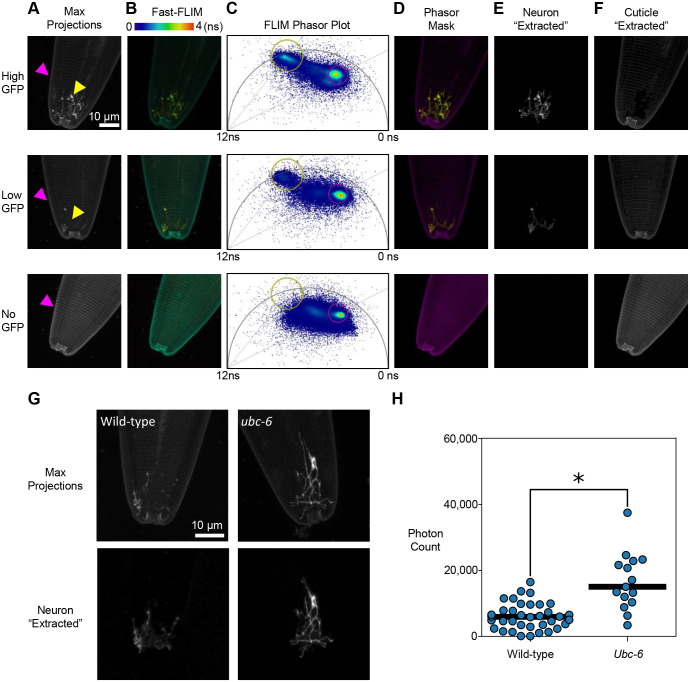
**Phasor-FLIM masking to isolate GFP fluorescence from autofluorescence.** (A) maximum intensity projections of *C. elegans* head showing high, low, or no GFP fluorescence in the AWA neuron excited with a 473 nm laser line. The cuticle and neuron are indicated with magenta and yellow arrows, respectively. Images are scaled to avoid saturation of the GFP signal. Genotypes imaged were *ubc-7* (*gk857464*); *kyIs53* (top), *kyIs53* (middle), N2 (bottom). (B) Fast-FLIM images displayed with a time-coded lookup table (LUT) showing longer lifetime GFP fluorescence (green/yellow) and shorter lifetime autofluorescence (cyan). (C) Phasor plots that correspond to the Fast-FLIM images in column (B). The yellow circle encapsulates the phasor space corresponding to GFP fluorescence. The magenta circle encapsulates the phasor space corresponding to cuticle autofluorescence. The horizontal axis of the phasor plot represents the *g* component, while the vertical axis represents the *s* component. The single exponential lifetime for pixels that fall on the universal semi-circle are between 0 and 12 ns, which is based upon the laser repetition rate of 80 mHz. (D) False colored images showing the GFP (yellow) and cuticle autofluorescence (magenta) regions of the phasor plots shown in column C. (E,F) Maximum intensity projections of the GFP pixels (column E) and cuticle autofluorescence pixels (column F) as defined in column D. These results were generated from three independent animals imaged in a single imaging session. (G) Representative images of Phasor-FLIM masking applied to kyIs53 worms in a wild-type and *ubc-6* mutant background. (H) GFP signal quantified and compared between the wild-type and *ubc-6* mutant background. 36 wild-type animals were imaged over six imaging sessions; 15 *ubc-*6 mutant animals were imaged over three imaging sessions. Normality of each group was checked using a Shapiro-Wilk test (wild-type: *P*=0.18, *ubc-6*: *P*=0.61). Groups were then compared using a two-tailed Student's *t*-test, which indicated a significant difference (*P*<0.001).

Our spectral analysis agrees with the well-documented interference from autofluorescence in the *C. elegans* gut and the cuticle, which can make it difficult to quantify weak GFP signals (Chalfie et al., 1994; Hermann et al., 2005; Monici, 2005; Morris et al., 2018; Pincus et al., 2016; Teuscher and Ewald, 2018). Although the GFP and autofluorescence spectra overlap, we hypothesized that their lifetimes could be resolved, which would allow GFP intensity to be measured even in the presence of background autofluorescence. To test this possibility, we imaged animals expressing P*odr*-10::ODR-10::GFP, which is an odorant receptor protein that localizes to ciliated sensory neurons at the anterior end of the animal (Ryan et al., 2014; Sengupta et al., 1996)*.* Regardless of the presence of the fluorescent transgene, we found that excitation using the 473 nm laser led to cuticle autofluorescence (Fig. 2A, magenta arrowhead). In animals with high levels of ODR-10::GFP expression, the GFP signal could be discerned over the cuticle autofluorescence (Fig. 2A, top row, yellow arrowhead). In animals whose ODR-10::GFP levels were relatively low, the cuticle autofluorescence obscured the GFP fluorescence (Fig. 2A, middle row, yellow arrowhead). No GFP fluorescence was seen in animals lacking the ODR-10::GFP transgene (Fig. 2A, bottom row).

To differentiate between cuticle autofluorescence and GFP fluorescence, we characterized each using Fast-FLIM (i.e. average photon arrival time, Fig. 2B). Regardless of expression level, the average photon arrival time of ODR-10::GFP was approximately 2.5 ns (Fig. 2B, top and middle rows), and the average photon arrival time of cuticle autofluorescence was approximately 1.3 ns (Fig. 2B, all rows). This suggested that FLIM could be used to separate these spectrally similar signals. However, Fast-FLIM has limited utility because it does not distinguish heterogeneous lifetimes within a single pixel. To more fully characterize cuticle and ODR-10::GFP fluorescence, we used Phasor-FLIM (Fig. 2C). On phasor plots, the ODR-10::GFP signal is located near the unit semi-circle at approximately 2.5 ns (based upon the 80 mHz repetition rate of our laser), which is indicative of a single, well-defined lifetime (Fig. 2C, yellow circle; Digman et al., 2008). In contrast, the cuticle signal is located in the interior of the unit semi-circle as a right shifted, tight cluster, which is indicative of shorter, heterogeneous lifetimes (Fig. 2C, magenta circle). These results suggest that cuticle autofluorescence arises from green fluorophores with complex decay profiles that can be spatially resolved in phasor space from the single component ODR-10::GFP. Indeed, when phasor masking is applied to these images, both bright and dim ODR-10::GFP signal can be faithfully ‘extracted’ from cuticle autofluorescence in live animals (Fig. 2D-F, top and middle rows). To demonstrate the biological usefulness of phasor masking, we used this process to characterize how the genetic mutation of the putative E2 ubiquitin ligase, *ubc-6,* affects ODR-10::GFP abundance. *ubc-6* is a highly conserved eukaryotic gene that participates in ER-associated degradation (Christianson and Carvalho, 2022; Weber et al., 2016), but no previous studies have implicated it in olfactory receptor maintenance. We found that a deletion in the *ubc-6* gene results in a 2.7-fold increase in the ciliary accumulation of ODR-10::GFP (average total photon counts for wild-type=6075, *ubc-6* mutant*=*16,657, Student's *t*-test *P*-value <0.001; Fig. 2G,H).

**Fig. 3.**
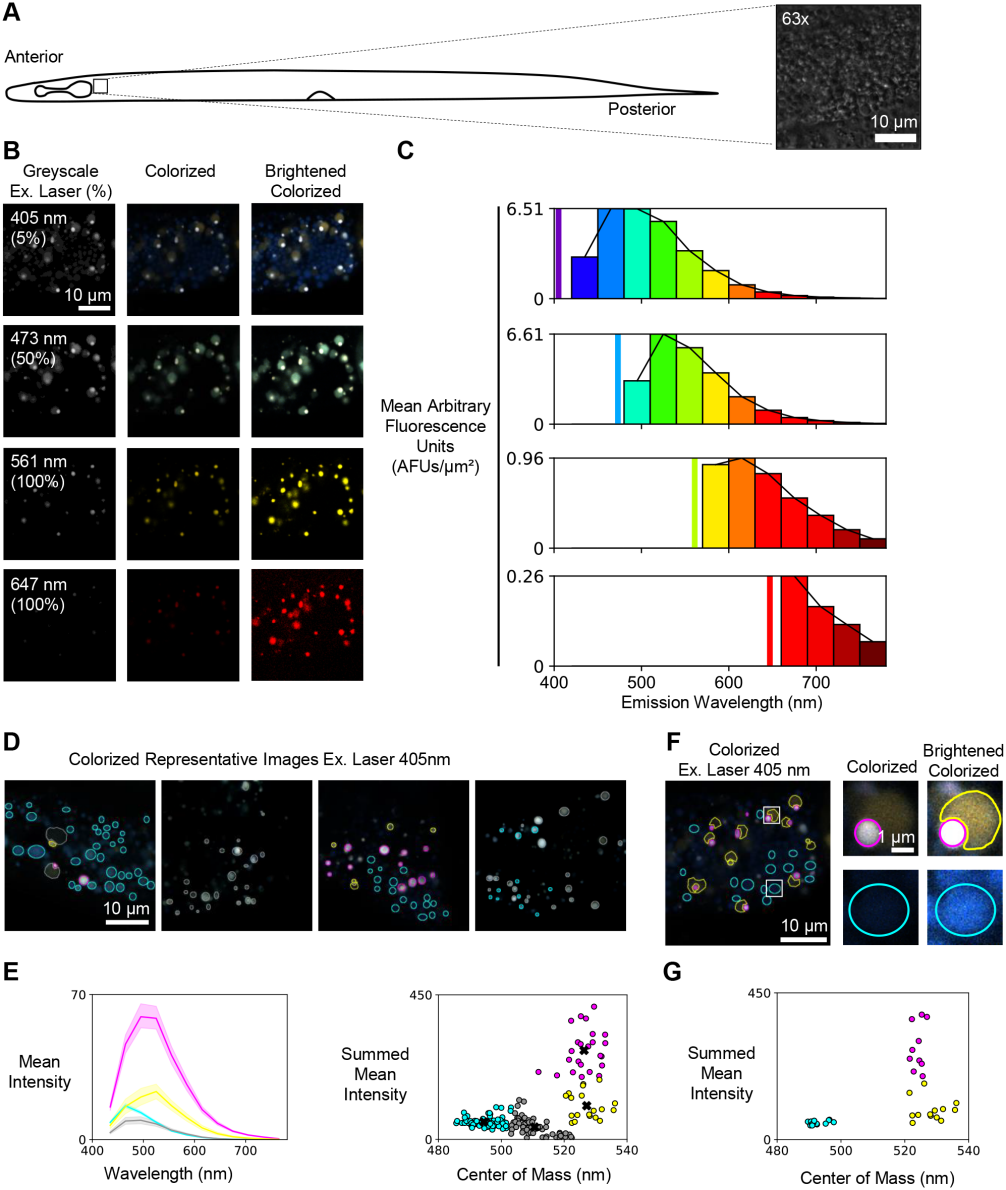
**Autofluorescent granules have spectrally distinct populations and regions.** (A) Schematic representation of a 3-day-old *C. elegans* and a region of the upper intestine captured at 63× magnification (inset) using DIC. (B) Images of a single plane in the intestine region (A, inset) showing autofluorescence emission after excitation with the following laser lines: 405 nm (emission 420-780 nm), 473 nm (emission 480-780 nm), 561 nm (emission 570-780 nm), and 647 nm (emission 660-780 nm). The left column shows total photon count as a greyscale image with the excitation wavelength and relative laser power is reported as a percentage. The middle column shows colorized spectral images. The right column shows brightened colorized spectral images. (C) The spectral profiles for each colorized spectral image in panel B. (D) Colorized spectral images of granules stimulated with the 405 nm laser line. Outlines indicate regions of interest that were quantified across the entire emission spectrum. (E) Left, the average emission of 187 granules across six images from six different animals outlined within the field of view, as shown in D (line color refers to the outline and the shading around the central line shows standard deviation from the average). Right, Kmeans++ clustering identifies four clusters of granules based on their spectral center of mass and summed mean intensity. (F) Colorized spectral images of granules from panels A and B that were stimulated with the 405 nm laser line. (G) Kmeans++ analysis showing that granules and sub-regions of granules are distributed across three of the four clusters identified in E.

Our results establish that Phasor-FLIM can separate problematic cuticle autofluorescence from GFP fluorescence in dim ciliated neurons located in the head of the animal. Next, we investigated whether spectral emission scanning and FLIM could be combined to differentiate between populations of gut autofluorescence in the anterior of the animal (Fig. 3A), which is known to result from a heterogeneous collection of subcellular lysosome-related gut granules (Hermann et al., 2005; Morris et al., 2018). Similar to our lower resolution spectral analysis (Fig. 1), the anterior gut produced heterogeneous emission spectra from individual granules that was most strongly stimulated with the 405 nm and 473 nm laser lines (Fig. 3B,C). To more fully characterize the heterogeneity, we applied K means clustering to spectral profiles of individual granules produced by 405 nm excitation. Specifically, we used the summed mean emission intensity across all wavelengths (i.e. brightness) as one component and the intensity weighted center of the emission peak (i.e. center of mass) as the second component. The clustering analysis revealed four robust populations (Fig. 3D,E, Fig. S3). The brightest population had a center of mass at approximately 525 nm (Fig. 3D,E, magenta) and included both isolated granules and granules that overlap with a larger, dimmer population (Fig. 3F,G, yellow). There were two additional relatively dim populations with centers of mass at approximately 495 nm and 510 nm (Fig. 3D-G, cyan and grey, respectively). These results demonstrate that high spatial resolution emission scanning can be combined with unbiased clustering approaches to phenotype spectrally distinct granules.

**Fig. 4.**
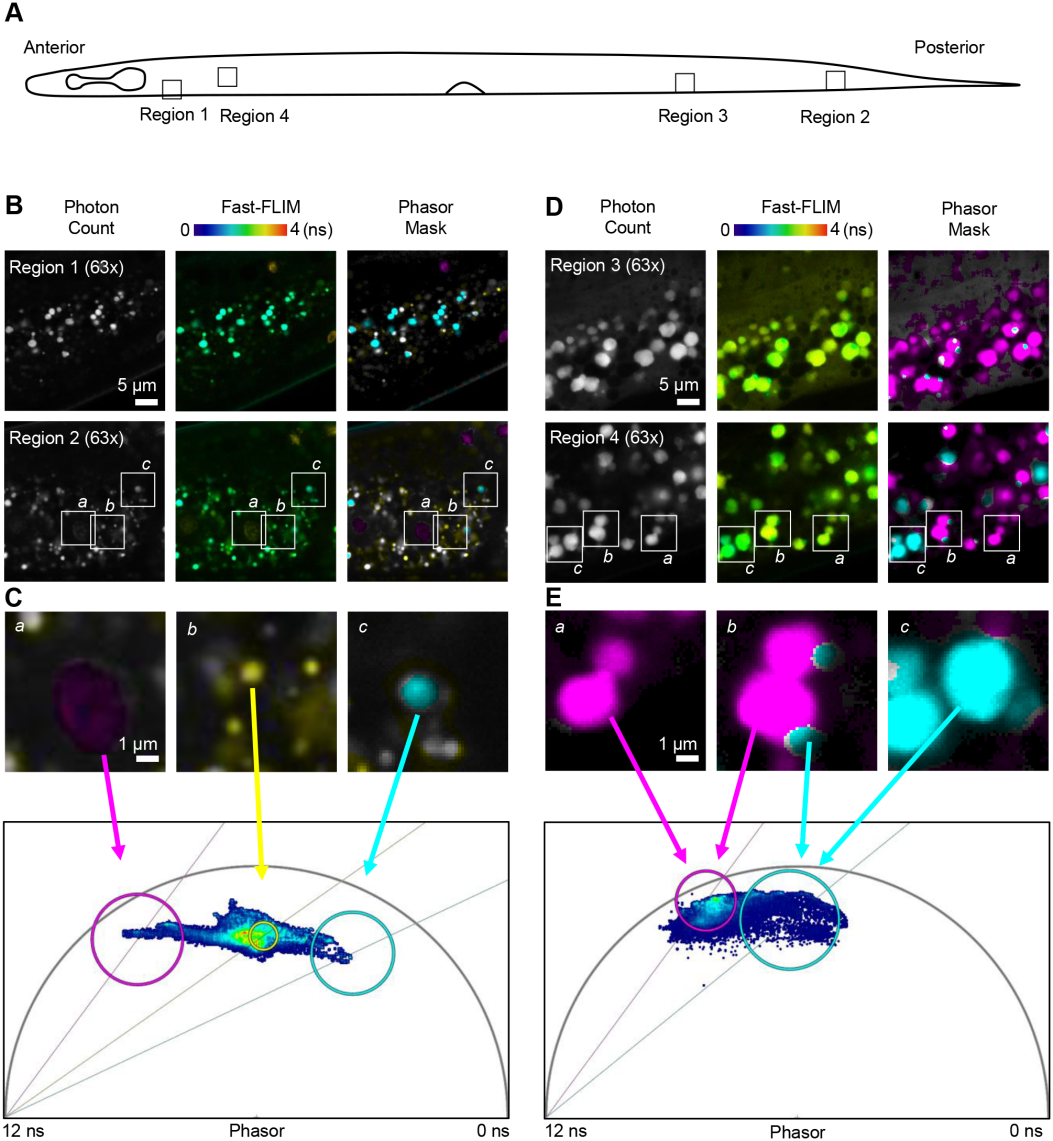
**Sub-populations of gut granules have distinct multi-exponential lifetimes and regions with distinct fluorescent lifetimes.** (A) A schematic representation of a 3-day-old *C. elegans.* Four gut regions were imaged with high spatial resolution FLIM (white boxes, regions 1-4). (B) Photon count images (left column), time-coded Fast-FLIM images (middle column), and photon count images with phasor overlay (phasor mask, right column) of a single plane in regions 1 and 2. The color scale for lifetime value is located above the column of Fast-FLIM images. (C) Zoomed images of granules with distinct multi-exponential lifetimes identified at the regions indicated in the phasor plot below (*a*, magenta; *b*, yellow; *c*, cyan). (D) Photon count images (left column), time-coded Fast-FLIM images (middle column), and photon count images with phasor overlay (phasor mask, right column) of a single plane in regions 3 and 4. The color scale for lifetime value is located above the column of Fast-FLIM images. (E) Zoomed images of granules composed of a single lifetime (*a*, magenta only and *c*, cyan only) and two examples of granules composed of two lifetimes (*b*, magenta and cyan). These results are representative of similar results that were replicated in four different animals across four different imaging sessions on four separate days.

Because GFP fluorescence could be separated from spectrally similar cuticle autofluorescence using FLIM (Fig. 2), we were curious whether FLIM could also reveal different subpopulations of gut granules. To test this, we analyzed fluorescence lifetime in several anterior and posterior regions of the gut using a 473 nm excitation laser (Fig. 4A). The photon count (i.e. intensity) images revealed granules with a range of intensities. These included both homogenous granules with uniform intensity and granules that appeared to have multiple compartments (Fig. 4B,D, left column; Fig. S4). Intriguingly, some of these granules could be visually distinguished via Fast-FLIM (Fig. 4B,D, middle column; Fig. S4). We analyzed phasor plots to further understand the nature of the different fluorescent lifetime populations. Phasor analysis revealed three distinct subpopulations of multi-component autofluorescence (i.e. located in the interior of the phasor plot) that originated from spatially distinct gut particles (Fig. 4B, right column; magenta, *a*; yellow, *b*; and cyan, *c*). Generally, the magenta phasor population (Fig. 4C, *a*) was composed of relatively large, low intensity granules that were sparsely distributed across the gut. In contrast, the yellow and cyan populations included both well-defined granules and diffuse regions of autofluorescence that lacked clear boundaries (Fig. 4C, *b* and *c*). In addition, we observed individual granules that could be separated into spatially distinct areas of the phasor plot (Fig. 4D, right column). Specifically, within a mixed population, some – but not all – granules could be separated into more than one lifetime (compare Fig. 4E, *a-c*, magenta, *a*; magenta and cyan, *b*; and cyan only, *c*). Collectively, these results demonstrate that spatially distinct gut granule autofluorescence can be more fully characterized in a label-free manner through a combination of high-resolution spectral and Phasor-FLIM analysis.

FLIM is advancing as a useful tool to overcome challenging microscopy problems (Datta et al., 2020) that include label-free analysis of autofluorescent cell structures and molecules (Blacker et al., 2014; Ouyang et al., 2021), biochemical characterization of the solvent surrounding known fluorophores (Llères et al., 2017), and distinguishing spectrally similar fluorophores (Scipioni et al., 2021). Here, we used FLIM to facilitate traditionally problematic quantification of dim GFP signal within sub-micron scale cell structures (i.e. sensory cilia) that are obscured by the green component of cuticle autofluorescence [Fig. 2 and as seen in Sepulveda et al. (2023); Wang et al. (2015)]. Compared to prior techniques, our FLIM method has the major benefit of not requiring the re-engineering of strains with different fluorescent reporters (Heppert et al., 2016) or purchasing an extensive array of overlapping bandpass filters (Morris et al., 2018). Although FLIM setups themselves can be costly and technically complex, as commercial systems become more common it is expected that using FLIM to isolate and quantify GFP signal will become more accessible. Moreover, because cuticle autofluorescence (Fig. 2) and gut autofluorescence (Fig. 4) exhibit complex decay profiles (i.e. they map to the interior of the phasor plot), the autofluorescence elimination approach described in this manuscript should be able to distinguish autofluorescence from any fluorescent protein that exhibits mono-exponential decay.

In addition, we have used FLIM to reveal sub-populations of autofluorescent lysosome-related organelles (gut granules) that can be separated based upon lifetime differences alone. This complements recent analytical approaches that combine Nile Red staining with two-photon FLIM to differentiate gut granules with distinct lipid populations (Chen et al., 2023). We have also used excitation/emission scanning to identify spectrally distinct subpopulations of gut granules that are uniquely excited at 405 nm. In the future, it will be important to identify how these spectrally distinct gut granules relate to those that can be distinguished via FLIM alone. However, this will require a pulsed ultraviolet (UV) laser to simultaneously excite the spectrally distinct population and perform time-correlated single photon counting, which is not presently available on commercial FLIM instruments.

*C. elegans* gut granules are an established model for understanding nutrient trafficking and metabolism. While many studies have focused on the endocytic pathways that underlie gut granule maturation, it is becoming clear that age and nutritional states can affect the physical, biochemical, and visual properties of gut granules (Chen et al., 2018; Chen et al., 2023; Hermann et al., 2005; Roh et al., 2012). For example, when animals are reared in excess zinc, gut granules form with a bilobed morphology (Mendoza et al., 2024; Roh et al., 2012). Because only one of the lobes consistently contains high concentrations of zinc, these granules are physiologically and spatially asymmetric (Mendoza et al., 2024). Our observation of some gut granules that contain fluorescence with more than one lifetime species is particularly reminiscent of these bilobed granules (Mendoza et al., 2024; Roh et al., 2012), though we did not rear animals on artificially high zinc concentrations.

The gut granules that we describe in this manuscript appear to represent spectrally defined categories, but they are not homogeneous with respect to representation and localization (Figs 3 and 4, Figs S3 and S4). This heterogeneity could arise from several aspects of *C. elegans* biology. First, because our samples were intentionally unlabeled, we did not attempt to identify different classifications of organelles. That is, it is possible that some of the granules that appear in our images represent lysosomes, endosomes, or other compartments derived from the endomembrane system. In addition, lysosome related organelles (LROs) undergo changes within developing and aging *C. elegans*. For example, protein markers for LROs can be detected during late embryonic and early larval stages (Hermann et al., 2005), but changes in lipid accumulation in LROs continue later, as the animals reach reproductive maturity and yolk proteins and lipids are transferred to maturing oocytes (Komura et al., 2021; Schroeder et al., 2007). Birefringence in LROs also increases as animals age (Komura et al., 2021). While we imaged animals after their final molt (from L4 larvae to adult animals), it is possible that our imaging captured granules that were in different stages of maturity. Finally, LROs are increasingly recognized as centers of metabolic regulation and metabolite storage. In particular, LROs can accumulate zinc (Roh et al., 2012), copper (Chun et al., 2017), and anthranilic acid glucosyl ester (downstream of kynurenine pathway, reviewed in Coburn and Gems, 2013). Importantly, even in animals experiencing a high metabolic input (for example, high levels of zinc), changes in gut granules labeling, size, and shape are heterogeneous (Roh et al., 2012). In our own analysis, we found some heterogeneity in spectral and FLIM profiles depending on where images were located (Figs S3 and S4). Overall, our data may be capturing the existing heterogeneity in the gut granule populations. Future experiments in animals lacking LROs, for example *glo-1* mutants (Hermann et al., 2005; Rabbitts et al., 2008), could be used to parse the precise identity of the granules we have described. In addition, monitoring and/or intentionally modifying metabolic inputs could drive gut granules to more heterogeneous spectral profiles.

Our imaging data show that autofluorescence can be masked to remove signal that may interfere with fluorescence imaging. With respect to understanding the biology of endomembrane trafficking in the gut, this is important because gut granule autofluorescence complicates the imaging and analysis of particles as they mature (Rabbitts et al., 2008; Voss et al., 2020). Previously, researchers depended on specific filter sets and protocols to try to remove background autofluorescence (Teuscher and Ewald, 2018). Alternatively, lipophilic or metal-binding dyes have been effective at boosting the signal of organelles of interest (Mendoza et al., 2024; Sepulveda et al., 2023). Recently, gut granule stores of heme have been assessed in a dye-free assay using transient absorption microscopy, but this relies specifically on the chemical signature of heme (Chen et al., 2018). Our FLIM data suggest that biochemical differences within subpopulations, and even individual gut granules, could be differentiated without the need for labeling or knowledge of precise chemical differences. Collectively, our results demonstrate that high spatial resolution spectral scanning combined with Phasor-FLIM is a useful tool to overcome challenging live imaging problems in *C. elegans* biology.

## MATERIALS AND METHODS

### *C. elegans* strains used in this study

N2 (Bristol), *kyIs53* (P*odr-10::ODR-10::*GFP), *kyIs53*; *ubc-7* (*gk857464*), *kyIs53*; *ubc-6* (*gk3799 gk5313[loxP*]). *C. elegans* were maintained according to accepted protocols (Brenner, 1974; Meneely et al., 2019).

### Preparing slides

Animals were grown at 21.5°C on nematode growth media (NGM) spotted with OP-50 *E. coli*. Animals were age synchronized by dissolving gravid animals and allowing the remaining eggs to hatch on NGM plates (Porta-de-la-Riva et al., 2012). Age synchronized young adult animals were paralyzed in an 8 µl droplet of 30 mg/ml 2,3-butanedione monoxime on a glass coverslip for 10 min. A 2% agarose pad was used to hold the fully immobilized animals for imaging.

### Microscope description

All imaging was performed on a Leica Stellaris 8 equipped with an 80 mHz pulsed white light laser that is tunable in 1 nm increments from 440-790 nm, a 405 nm diode (non-pulsed) laser, and five HyD detectors with dispersion-based spectral scanning from 410-850 nm. The microscope is equipped with a 63×1.4 NA oil objective, a 63×1.2 NA water objective, a 40×1.4 NA oil objective, 25×0.95 NA water objective, 20×0.75 NA dry objective, 10×0.4 NA dry objective. The microscope is controlled by LasX software that includes the Falcon FLIM module (including phasor analysis), Lightning deconvolution, and TauSense. For all imaging experiments, the 405 diode and white light laser were both turned on 45 min before data were collected to allow them to warm up. All laser intensities reported in this manuscript are relative – laser power at the sample was not determined.

### Spectral scans of entire *C. elegans*

To capture emission profiles of the entire animal, the 20×/0.75 objective lens was used with a digital zoom of 4.44 to create a tile scan of the animal with a 256.19 nm pixel size. To capture emission profiles of gut granules, the 63×/1.4 oil objective lens was used with a digital zoom of 5.26 to create single images with a pixel size of 68.65 nm. The focal plane for the emission scanning was approximately midway through the animal. Four commonly used excitation wavelengths (405 nm, 473 nm, 561 nm, and 647 nm) were used to create emission profiles in 30 nm increments from 420-780 nm (405 nm excitation), 480-780 nm (473 nm excitation), 570-780 nm (561 nm excitation), and 660-780 nm (647 nm excitation). The spectral scan information is stored in image stacks where each slice contains the intensity information for a 30 nm band of the emission profile (see Fig. S1B-D for an example of spectral image stack).

### Colorized spectral images and spectral plots

To create spectral plots of the entire animal (Fig. 1D) or of individual gut regions (Fig. 3C) the average intensity per unit area was calculated for regions of interest and plotted against the center of the respective emission band. To colorize the spectral image data, the slice corresponding to each emission band was converted to an RGB color corresponding to the average wavelength for that emission band [e.g. 435 nm (blue) for the 420-450 nm band and 645 nm (red) for the 630-660 nm band]. These RGB images were then summed to produce a fully colorized image. For example, if a region of interest had strong emission in the blue, green, and red bands, the summed colorized image would appear white, but if there was strong emission in green and red bands, the summed colorized image would appear yellow. The same steps were followed for ‘brightened colorized’ images, except the contrast was adjusted to saturate ≤0.125% of pixels before making the figure. To characterize gut granule emission, the R program Kmeans++ was used to cluster individual gut granules based upon the summed mean intensity (i.e. brightness) and the center of mass (i.e. color) of their spectral profiles (Fig. 3D-G and Fig. S3). The gut granules were manually outlined in FIJI prior to Kmeans++ clustering.

### General procedure for separating cuticle autofluorescence from GFP fluorescence

Images were acquired as Z-stacks with a 1 µm step size using a 63×/1.40 oil objective, a zoom of 4, and a resolution of 512×512 pixels, which leads to a 90 nm pixel size. The scan speed was set to 600 Hz with four-line repetitions and the 488 nm laser set to 100% power. The acquisition was conducted using LasX FALCON/FLIM, which sets the emission detector to single photon counting mode and synchronizes the electronics to operate as a time-correlated single photon counter. Photon count images represent the total number of photons collected at each pixel (i.e. intensity). Fast-FLIM images represent the average photon arrival time at each pixel. Phasor analysis was performed with the following settings: Pixel Binning: 1, Harmonic: 1, Threshold: 15 photons, Median Filter Radius: 11 pixels. After identifying the phasor space that contained the GFP signal and the autofluorescence signal, a circular phasor mask was created to encapsulate the appropriate area. After a region of the phasor plot was selected in LasX, the corresponding image pixels were exported as a mask. To mask GFP, a 50-pixel circle centered at 2.561 ns was used. To mask cuticle autofluorescence, a 30-pixel circle centered at 1.017 ns was used. The mask images were imported into ImageJ where all pixels outside of the mask were set to 0.

### Quantification of ODR-10::GFP accumulation in AWA cilia

Images of wild-type and *ubc-6* mutant animals were obtained with the following settings: Objective: 63×/1.40 oil, resolution: 512×512, zoom: 4, pixel size: 90 nm, step size: 1 µm, scan speed: 600 Hz, line repetitions: 4, laser: 488 nm excitation with 50% intensity. FLIM characterization was performed with the following settings: pixel binning: 1, harmonic: 1, threshold: 7 photons, median filter radius: 19. GFP signal was extracted as described above. The resulting GFP images were processed using a FIJI macro found here: https://github.com/heinohv/Dahlberg-Lab/blob/main/photon_measure.ijm.

### FLIM analysis of gut granules

The images were captured with the following settings: Objective: 63×/1.40 oil, resolution: 512×512, zoom: 5.26, pixel size: 69 nm, scan speed: 600 Hz, line repetitions: 8, laser: 473 nm excitation with 10% intensity. FLIM characterization and export was performing with the following settings: pixel binning: 2, hamonic: 1, threshold 20-100 photons, median filter radius: 11. To characterize different granules based on fluorescent lifetime, phasor plots were manually scanned to identify gut granules, or parts of gut granules, whose autofluorescence could be mapped back to discrete regions of phasor space.

## Supplementary Material

10.1242/biolopen.060613_sup1 Supplementary information

## References

[BIO060613C1] Bao, Z., Murray, J. I., Boyle, T., Ooi, S. L., Sandel, M. J. and Waterston, R. H. (2006). Automated cell lineage tracing in Caenorhabditis elegans. *Proc. Natl Acad. Sci. USA* 103, 2707-2712. 10.1073/pnas.0511111103 16477039 PMC1413828

[BIO060613C2] Bhattacharjee, A., Datta, R., Gratton, E. and Hochbaum, A. I. (2017). Metabolic fingerprinting of bacteria by fluorescence lifetime imaging microscopy. *Sci. Rep.* 7, 3743. 10.1038/s41598-017-04032-w 28623341 PMC5473825

[BIO060613C3] Blacker, T. S., Mann, Z. F., Gale, J. E., Ziegler, M., Bain, A. J., Szabadkai, G. and Duchen, M. R. (2014). Separating NADH and NADPH fluorescence in live cells and tissues using FLIM. *Nat. Commun.* 5, 1-9. 10.1038/ncomms4936 PMC4046109 24874098

[BIO060613C4] Brenner, S. (1974). THE GENETICS OF *CAENORHABDITIS ELEGANS*. *Genetics* 77, 71-94. 10.1093/genetics/77.1.71 4366476 PMC1213120

[BIO060613C5] Chalfie, M., Tu, Y., Euskirchen, G., Ward, W. W. and Prasher, D. C. (1994). Green fluorescent protein as a marker for gene expression. *Science* 263, 802-805. 10.1126/science.8303295 8303295

[BIO060613C6] Chen, A. J., Yuan, X., Li, J., Dong, P., Hamza, I. and Cheng, J.-X. (2018). Label-free imaging of heme dynamics in living organisms by transient absorption microscopy. *Anal. Chem.* 90, 3395-3401. 10.1021/acs.analchem.7b05046 29401392 PMC5972037

[BIO060613C7] Chen, W.-W., Tang, W., Hamerton, E. K., Kuo, P. X., Lemieux, G. A., Ashrafi, K. and Cicerone, M. T. (2023). Identifying lipid particle sub-types in live Caenorhabditis elegans with two-photon fluorescence lifetime imaging. *Front. Chem.* 11, 1161775. https://www.frontiersin.org/articles/10.3389/fchem.2023.1161775 10.3389/fchem.2023.1161775 37123874 PMC10137682

[BIO060613C8] Christianson, J. C. and Carvalho, P. (2022). Order through destruction: How ER–associated protein degradation contributes to organelle homeostasis. *EMBO J.* 41, e109845. 10.15252/embj.2021109845 35170763 PMC8922271

[BIO060613C9] Chun, H., Sharma, A. K., Lee, J., Chan, J., Jia, S. and Kim, B.-E. (2017). The intestinal copper exporter CUA-1 is required for systemic copper homeostasis in *Caenorhabditis elegans*. *J. Biol. Chem.* 292, 1-14. 10.1074/jbc.M116.760876 27881675 PMC5217669

[BIO060613C10] Chung, S. H., Sun, L. and Gabel, C. V. (2013). In vivo neuronal calcium imaging in C. elegans. *J. Vis. Exp.* 74, e50357. 10.3791/50357 PMC3653678 23603812

[BIO060613C11] Coburn, C. and Gems, D. (2013). The mysterious case of the C. elegans gut granule: Death fluorescence, anthranilic acid and the kynurenine pathway. *Front. Genet.* 4, 151. 10.3389/fgene.2013.00151 23967012 PMC3735983

[BIO060613C12] Corsi, A. K., Wightman, B. and Chalfie, M. (2015). A transparent window into biology: a primer on Caenorhabditis elegans. *Genetics* 200, 387-407. 10.1534/genetics.115.176099 26088431 PMC4492366

[BIO060613C13] Datta, R., Heaster, T. M., Sharick, J. T., Gillette, A. A. and Skala, M. C. (2020). Fluorescence lifetime imaging microscopy: Fundamentals and advances in instrumentation, analysis, and applications. *J. Biomed. Opt.* 25, 071203. 10.1117/1.JBO.25.7.071203 32406215 PMC7219965

[BIO060613C14] Digman, M. A., Caiolfa, V. R., Zamai, M. and Gratton, E. (2008). The phasor approach to fluorescence lifetime imaging analysis. *Biophys. J.* 94, L14-L16. 10.1529/biophysj.107.120154 17981902 PMC2157251

[BIO060613C15] Eichel, K., Uenaka, T., Belapurkar, V., Lu, R., Cheng, S., Pak, J. S., Taylor, C. A., Südhof, T. C., Malenka, R., Wernig, M. et al. (2022). Endocytosis in the axon initial segment maintains neuronal polarity. *Nature* 609, 128-135. 10.1038/s41586-022-05074-5 35978188 PMC9433327

[BIO060613C16] El Mouridi, S., Alkhaldi, F. and Frøkjær-Jensen, C. (2022). Modular safe-harbor transgene insertion for targeted single-copy and extrachromosomal array integration in Caenorhabditis elegans. *G3 (Bethesda)* 12, jkac184. 10.1093/g3journal/jkac184 35900171 PMC9434227

[BIO060613C17] Gallrein, C., Iburg, M., Michelberger, T., Koçak, A., Puchkov, D., Liu, F., Ayala Mariscal, S. M., Nayak, T., Kaminski Schierle, G. S. and Kirstein, J. (2021). Novel amyloid-beta pathology *C. elegans* model reveals distinct neurons as seeds of pathogenicity. *Prog. Neurobiol.* 198, 101907. 10.1016/j.pneurobio.2020.101907 32926945

[BIO060613C18] Gonzalez Pisfil, M., Nadelson, I., Bergner, B., Rottmeier, S., Thomae, A. W. and Dietzel, S. (2022). Stimulated emission depletion microscopy with a single depletion laser using five fluorochromes and fluorescence lifetime phasor separation. *Sci. Rep.* 12, 14027. 10.1038/s41598-022-17825-5 35982114 PMC9388687

[BIO060613C19] Hendler-Neumark, A., Wulf, V. and Bisker, G. (2021). In vivo imaging of fluorescent single-walled carbon nanotubes within C. elegans nematodes in the near-infrared window. *Mater. Today Bio.* 12, 100175. 10.1016/j.mtbio.2021.100175 PMC8649898 34927042

[BIO060613C20] Heppert, J. K., Dickinson, D. J., Pani, A. M., Higgins, C. D., Steward, A., Ahringer, J., Kuhn, J. R. and Goldstein, B. (2016). Comparative assessment of fluorescent proteins for in vivo imaging in an animal model system. *Mol. Biol. Cell* 27, 3385-3394. 10.1091/mbc.E16-01-0063 27385332 PMC5221575

[BIO060613C21] Hermann, G. J., Schroeder, L. K., Hieb, C. A., Kershner, A. M., Rabbitts, B. M., Fonarev, P., Grant, B. D. and Priess, J. R. (2005). Genetic analysis of lysosomal trafficking in Caenorhabditis elegans. *Mol. Biol. Cell* 16, 3273-3288. 10.1091/mbc.E05-01-0060 15843430 PMC1165410

[BIO060613C22] Jones, K. T. and Ashrafi, K. (2009). Caenorhabditis elegans as an emerging model for studying the basic biology of obesity. *Dis. Model. Mech.* 2, 224-229. 10.1242/dmm.001933 19407330 PMC2675801

[BIO060613C23] Komura, T., Yamanaka, M., Nishimura, K., Hara, K. and Nishikawa, Y. (2021). Autofluorescence as a noninvasive biomarker of senescence and advanced glycation end products in Caenorhabditis elegans. *NPJ Aging Mech. Dis.* 7, 12. 10.1038/s41514-021-00061-y 34099724 PMC8184826

[BIO060613C24] Laine, R. F., Sinnige, T., Ma, K. Y., Haack, A. J., Poudel, C., Gaida, P., Curry, N., Perni, M., Nollen, E. A. A., Dobson, C. M. et al. (2019). Fast fluorescence lifetime imaging reveals the aggregation processes of α-synuclein and polyglutamine in aging *Caenorhabditis elegans*. *ACS Chem. Biol.* 14, 1628-1636. 10.1021/acschembio.9b00354 31246415 PMC7612977

[BIO060613C25] Llères, D., Bailly, A. P., Perrin, A., Norman, D. G., Xirodimas, D. P. and Feil, R. (2017). Quantitative FLIM-FRET microscopy to monitor nanoscale chromatin compaction in vivo reveals structural roles of condensin complexes. *Cell Rep.* 18, 1791-1803. 10.1016/j.celrep.2017.01.043 28199849

[BIO060613C26] Lou, J., Scipioni, L., Wright, B. K., Bartolec, T. K., Zhang, J., Masamsetti, V. P., Gaus, K., Gratton, E., Cesare, A. J. and Hinde, E. (2019). Phasor histone FLIM-FRET microscopy quantifies spatiotemporal rearrangement of chromatin architecture during the DNA damage response. *Proc. Natl Acad. Sci. USA* 116, 7323-7332. 10.1073/pnas.1814965116 30918123 PMC6462080

[BIO060613C27] Ma, N., Digman, M. A., Malacrida, L. and Gratton, E. (2016). Measurements of absolute concentrations of NADH in cells using the phasor FLIM method. *Biomed. Opt. Express* 7, 2441-2452. 10.1364/BOE.7.002441 27446681 PMC4948605

[BIO060613C28] Malacrida, L., Ranjit, S., Jameson, D. M. and Gratton, E. (2021). The phasor plot: a universal circle to advance fluorescence lifetime analysis and interpretation. *Annu. Rev. Biophys.* 50, 575-593. 10.1146/annurev-biophys-062920-063631 33957055

[BIO060613C29] Mendoza, A. D., Dietrich, N., Tan, C.-H., Herrera, D., Kasiah, J., Payne, Z., Cubillas, C., Schneider, D. L. and Kornfeld, K. (2024). Lysosome-related organelles contain an expansion compartment that mediates delivery of zinc transporters to promote homeostasis. *Proc. Natl Acad. Sci. USA* 121, e2307143121. 10.1073/pnas.2307143121 38330011 PMC10873617

[BIO060613C30] Meneely, P. M., Dahlberg, C. L. and Rose, J. K. (2019). Working with worms: *Caenorhabditis elegans* as a model organism. *Curr. Protoc. Essent. Lab. Tech.* 19, e35. 10.1002/cpet.35

[BIO060613C31] Monici, M. (2005). Cell and tissue autofluorescence research and diagnostic applications. *Biotechnol. Annu. Rev.* 11, 227-256. 10.1016/S1387-2656(05)11007-2 16216779

[BIO060613C32] Morris, C., Foster, O. K., Handa, S., Peloza, K., Voss, L., Somhegyi, H., Jian, Y., Vo, M. V., Harp, M., Rambo, F. M. et al. (2018). Function and regulation of the Caenorhabditis elegans Rab32 family member GLO-1 in lysosome-related organelle biogenesis. *PLoS Genet.* 14, e1007772. 10.1371/journal.pgen.1007772 30419011 PMC6268011

[BIO060613C33] Ouyang, Y., Liu, Y., Wang, Z. M., Liu, Z. and Wu, M. (2021). FLIM as a promising tool for cancer diagnosis and treatment monitoring. *Nanomicro Lett.* 13, 133. 10.1007/s40820-021-00653-z 34138374 PMC8175610

[BIO060613C34] Pincus, Z., Mazer, T. C. and Slack, F. J. (2016). Autofluorescence as a measure of senescence in C. elegans: Look to red, not blue or green. *Aging (Albany NY)* 8, 889-898. 10.18632/aging.100936 27070172 PMC4931842

[BIO060613C35] Porta-de-la-Riva, M., Fontrodona, L., Villanueva, A. and Cerón, J. (2012). Basic Caenorhabditis elegans methods: synchronization and observation. *J. Vis. Exp.* 64, 4019. 10.3791/4019 PMC3607348 22710399

[BIO060613C36] Rabbitts, B. M., Ciotti, M. K., Miller, N. E., Kramer, M., Lawrenson, A. L., Levitte, S., Kremer, S., Kwan, E., Weis, A. M. and Hermann, G. J. (2008). *Glo-3*, a novel *Caenorhabditis elegans* gene, is required for lysosome-related organelle biogenesis. *Genetics* 180, 857-871. 10.1534/genetics.108.093534 18780725 PMC2567386

[BIO060613C37] Rashtchian, S., Youssef, K., Rezai, P. and Tabatabaei, N. (2021). High-speed label-free confocal microscopy of Caenorhabditis elegans with near infrared spectrally encoded confocal microscopy. *Biomed. Opt. Express* 12, 3607-3618. 10.1364/BOE.427685 34221682 PMC8221957

[BIO060613C38] Rodrigues, N. T. L., Bland, T., Borrego-Pinto, J., Ng, K., Hirani, N., Gu, Y., Foo, S. and Goehring, N. W. (2022). SAIBR: A simple, platform-independent method for spectral autofluorescence correction. *Development* 149, dev200545. 10.1242/dev.200545 35713287 PMC9445497

[BIO060613C39] Roh, H. C., Collier, S., Guthrie, J., Robertson, J. D. and Kornfeld, K. (2012). Lysosome-related organelles in intestinal cells are a zinc storage site in C. elegans. *Cell Metab.* 15, 88-99. 10.1016/j.cmet.2011.12.003 22225878 PMC4026189

[BIO060613C40] Ryan, D. A., Miller, R. M., Lee, K., Neal, S. J., Fagan, K. A., Sengupta, P. and Portman, D. S. (2014). Sex, age, and hunger regulate behavioral prioritization through dynamic modulation of chemoreceptor expression. *Curr. Biol.* 24, 2509-2517. 10.1016/j.cub.2014.09.032 25438941 PMC4254623

[BIO060613C41] Schroeder, L. K., Kremer, S., Kramer, M. J., Currie, E., Kwan, E., Watts, J. L., Lawrenson, A. L. and Hermann, G. J. (2007). Function of the Caenorhabditis elegans ABC transporter PGP-2 in the biogenesis of a lysosome-related fat storage organelle. *Mol. Biol. Cell* 18, 995-1008. 10.1091/mbc.e06-08-0685 17202409 PMC1805080

[BIO060613C42] Scipioni, L., Rossetta, A., Tedeschi, G. and Gratton, E. (2021). Phasor S-FLIM: A new paradigm for fast and robust spectral fluorescence lifetime imaging. *Nat. Methods* 18, 542-550. 10.1038/s41592-021-01108-4 33859440 PMC10161785

[BIO060613C43] Sengupta, P., Chou, J. H. and Bargmann, C. I. (1996). Odr-10 encodes a seven transmembrane domain olfactory receptor required for responses to the odorant diacetyl. *Cell* 84, 899-909. http://www.ncbi.nlm.nih.gov/pubmed/8601313. 10.1016/S0092-8674(00)81068-5 8601313

[BIO060613C44] Sepulveda, N. B., Chen, D. and Petrella, L. N. (2023). Moderate heat stress-induced sterility is due to motility defects and reduced mating drive in Caenorhabditis elegans males. *J. Exp. Biol.* 226, jeb245546. 10.1242/jeb.245546 37724024

[BIO060613C45] Szmacinski, H., Toshchakov, V. and Lakowicz, J. R. (2014). Application of phasor plot and autofluorescence correction for study of heterogeneous cell population. *J. Biomed. Opt.* 19, 046017. 10.1117/1.JBO.19.4.046017 24770662 PMC4000004

[BIO060613C46] Teuscher, A. C. and Ewald, C. Y. (2018). Overcoming autofluorescence to assess GFP expression during normal physiology and aging in Caenorhabditis elegans. *Bio. Protoc.* 8, e2940. 10.21769/BioProtoc.2940 PMC6067662 30073182

[BIO060613C47] Thomas, B. J., Wight, I. E., Chou, W. Y. Y., Moreno, M., Dawson, Z., Homayouni, A., Huang, H., Kim, H., Jia, H., Buland, J. R. et al. (2019). CemOrange2 fusions facilitate multifluorophore subcellular imaging in C. elegans. *PLoS ONE* 14, e0214257. 10.1371/journal.pone.0214257 30913273 PMC6435234

[BIO060613C48] Tian, L., Hires, S. A., Mao, T., Huber, D., Chiappe, M. E., Chalasani, S. H., Petreanu, L., Akerboom, J., McKinney, S. A., Schreiter, E. R. et al. (2009). Imaging neural activity in worms, flies and mice with improved GCaMP calcium indicators. *Nat. Methods* 6, 875-881. 10.1038/nmeth.1398 19898485 PMC2858873

[BIO060613C49] Voss, L., Foster, O. K., Harper, L., Morris, C., Lavoy, S., Brandt, J. N., Peloza, K., Handa, S., Maxfield, A., Harp, M. et al. (2020). An ABCG transporter functions in Rab localization and lysosome-related organelle biogenesis in *Caenorhabditis elegans*. *Genetics* 214, 419-445. 10.1534/genetics.119.302900 31848222 PMC7017009

[BIO060613C50] Wang, J., Kaletsky, R., Silva, M., Williams, A., Haas, L. A., Androwski, R. J., Landis, J. N., Patrick, C., Rashid, A., Santiago-Martinez, D. et al. (2015). Cell-specific transcriptional profiling of ciliated sensory neurons reveals regulators of behavior and extracellular vesicle biogenesis. *Curr. Biol.* 25, 3232-3238. 10.1016/j.cub.2015.10.057 26687621 PMC4698341

[BIO060613C51] Weber, A., Cohen, I., Popp, O., Dittmar, G., Reiss, Y., Sommer, T., Ravid, T. and Jarosch, E. (2016). Sequential Poly-ubiquitylation by specialized conjugating enzymes expands the versatility of a quality control ubiquitin ligase. *Mol. Cell* 63, 827-839. 10.1016/j.molcel.2016.07.020 27570077

[BIO060613C52] Yemini, E., Lin, A., Nejatbakhsh, A., Varol, E., Sun, R., Mena, G. E., Samuel, A. D. T., Paninski, L., Venkatachalam, V. and Hobert, O. (2021). NeuroPAL: a multicolor atlas for whole-brain neuronal identification in C. elegans. *Cell* 184, 272-288.e11. 10.1016/j.cell.2020.12.012 33378642 PMC10494711

