# Supplementary material for: Spectral scanning and fluorescence lifetime imaging microscopy (FLIM) enable separation and characterization of C. elegans autofluorescence in the cuticle and gut
Source: Biol Open. 2024 Dec 30;13(12):bio060613. doi: 10.1242/bio.060613 (PMC11708769; doi:10.1242/bio.060613)
Supplement: Supplementary information [file biolopen-13-060613-s1.pdf]

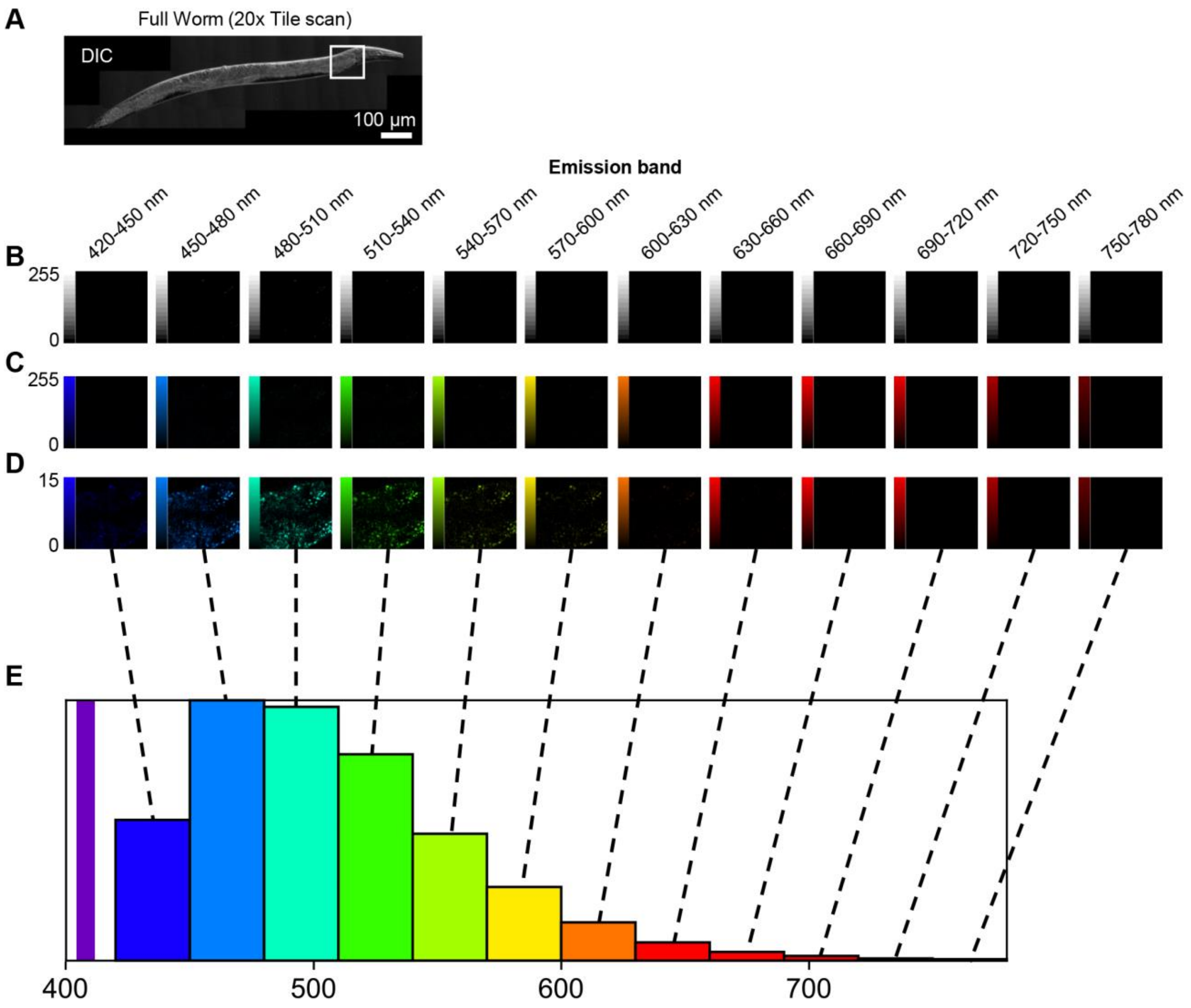

Fig. S1. Generation of colorized images based on spectral scanning. A) Image of the animal shown in Fig. 1. The white box indicates the portion of the animal used for this colorization example. B) XY Lambda (emission) stacks were split into 12 individual emission bins. C) Each emission bin was colorized based on the center of the emission bin. For example, for the 420-450 nm emission bin, the blue color LUT corresponds to different intensities of 435 nm light. D) Images from B are displayed with a narrow intensity range to make the dim signal more apparent. E) A generic spectral profile plot showing how each emission bin image corresponds to an emission bin average in the spectral profile plot.

Biology Open • Supplementary information

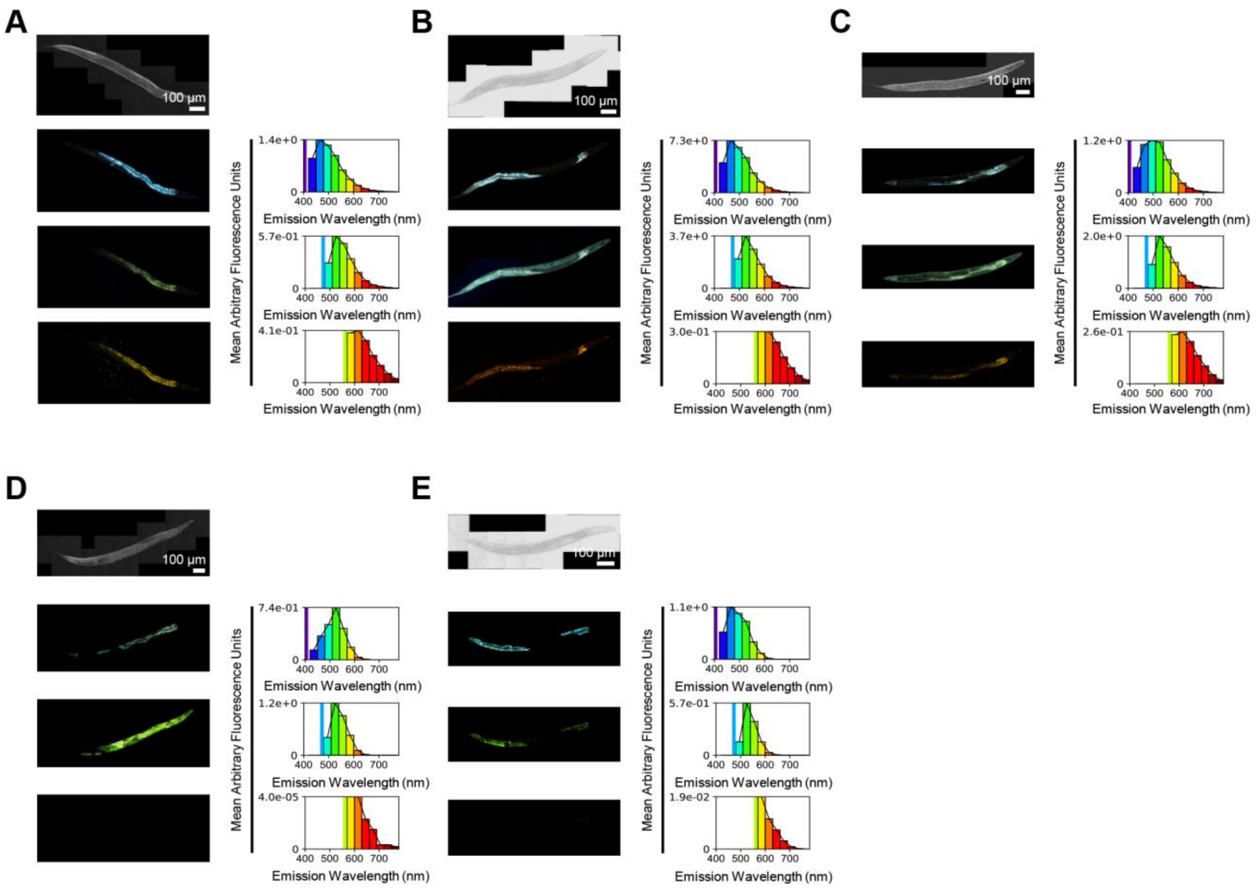

Fig. S2. Variable emission is stimulated by the 561 nm laser line. 3-day old *C. elegans* acquired using differential interference contrast (DIC) and the same spectral scanning approach described in Fig. 1. A-E) show consistent strong emission stimulated by the 405 nm and 473 nm laser lines. A-C) show weak but observable emission stimulated by the 561 nm laser line. D and E) show barely detectable emission stimulated by the 561 nm laser line. These results were generated from five independent animals imaged across two separate imaging sessions on two separate days.

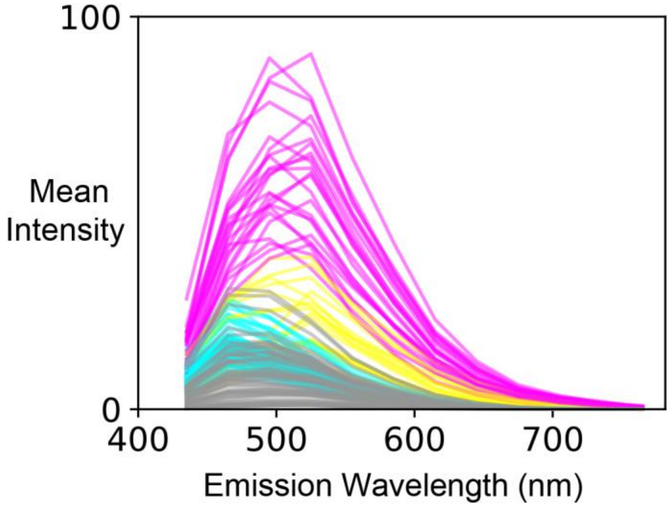

Fig. S3. Individual spectral profiles for spectrally distinct gut granules separated via Kmeans++ clustering. The individual spectral profiles for all 187 gut granules acquired from 6 separate animals that were analyzed in this study. The color coding of the spectral profiles was determined via Kmeans++ clustering. The average and standard deviation for each class of spectral profile is shown in Fig. 3E.

Biology Open • Supplementary information

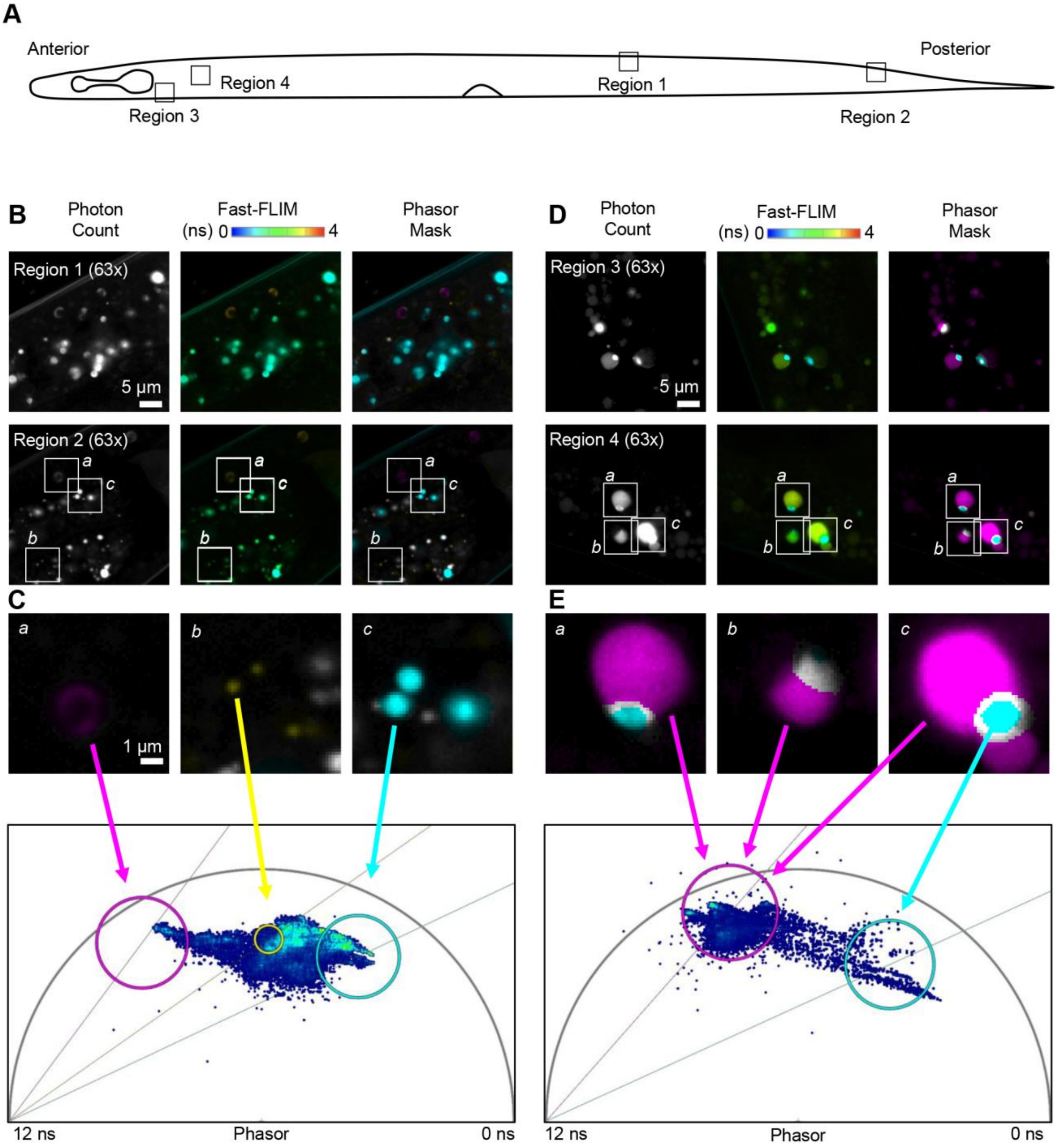

Fig. S4. Additional examples of autofluorescence with spatial heterogeneity in fluorescence lifetime throughout the gut of *C. elegans*. A) A schematic representation of a 3-day old *C. elegans.* As in Fig. 4, single planes in four gut regions were imaged with high spatial resolution FLIM (white boxes, regions 1-4). B) Photon count images (left column), time-coded Fast-FLIM images (middle column), and photon

Biology Open • Supplementary information

count images with phasor overlay (phasor mask, right column) of regions 1 and 2. The color scale for lifetime value is located above the column of Fast-FLIM images. C) Zoomed images of granules with distinct multi-exponential lifetimes identified at the regions indicated in the phasor plot below (*a*, magenta; *b*, yellow; *c*, cyan). D) Photon count images (left column), time-coded Fast-FLIM images (middle column), and photon count images with phasor overlay (phasor mask, right column) of regions 3 and 4. The color scale for lifetime value is located above the column of Fast-FLIM images. E) Zoomed images of granules composed of a single lifetime (*a,* magenta only and *c,* cyan only) and two examples of granules composed of two lifetimes (*b*, magenta and cyan). These results are representative of similar results that were replicated in four different animals across four different imaging sessions on four separate days.
